# Cloning and enhancing production of a detergent- and organic-solvent-resistant nattokinase from *Bacillus subtilis* VTCC-DVN-12-01 by using an eight-protease-gene-deficient *Bacillus subtilis* WB800

**DOI:** 10.1186/1475-2859-12-79

**Published:** 2013-09-10

**Authors:** Thao Thi Nguyen, Thi Dinh Quyen, Hoang Thanh Le

**Affiliations:** 1 Institute of Biotechnology, Vietnam Academy of Science and Technology, 18 Hoang Quoc Viet Road, Distr. Caugiay, Hanoi 10600, Vietnam; 2 Department of Biotechnology and Pharmacology, University of Science and Technology of Hanoi, Vietnam, 18 Hoang Quoc Viet Road, Distr. Cau giay, Hanoi 10600, Vietnam

**Keywords:** *Bacillus subtilis*, Nattokinase gene, High-level Expression, Characterization, Detergent- and organic-solvent resistance

## Abstract

**Background:**

Nattokinases/Subtilisins (EC 3.4.21.62) belong to the second large family of serine proteases, which gain significant attention and play important role in many biotechnology processes. Thus, a number of nattokinases/subtilisins from various *Bacillus* species, especially from *B. subtilis* strains, extensively have been investigated to understand their biochemical and physical properties as well as to improve the production for industrial application. The purpose of this study was to clone a nattokinase gene from *Bacillus subtilis* strain VTCC-DVN-12-01, enhance its production in *B. subtilis* WB800, which is deficient in eight extracellular proteases and characterize its physicochemical properties for potential application in organic synthesis and detergent production.

**Results:**

A gene coding for the nattokinase (Nk) from *B. subtilis* strain VTCC-DVN-12-01 consisted of an ORF of 1146 nucleotides, encoding a pre-pro-protein enzyme (30-aa pre-signal peptide, 76-aa pro-peptide and 275-aa mature protein with a predicted molecular mass of 27.7 kDa and pI 6.6). The nattokinase showed 98-99% identity with other nattokinases/subtilisins from *B. subtilis* strains in GenBank. Nk was expressed in *B. subtilis* WB800 under the control of *acoA* promoter at a high level of 600 mg protein per liter culture medium which is highest yield of proteins expressed in any extracellular-protease-deficient *B. subtilis* system till date. Nk was purified to homogeneity with 3.25 fold purification, a specific activity of 12.7 U/mg, and a recovery of 54.17%. The purified Nk was identified by MALDI-TOF mass spectrometry through three peptides, which showed 100% identity to corresponding peptides of the *B. subtilis* nattokinase (CAC41625). An optimal activity for Nk was observed at 65°C and pH 9. The nattokinase was stable at temperature up to 50°C and in pH range of 5–11 and retained more than 85% of its initial activity after incubation for 1 h. Mg^2+^ activated Nk up to 162% of its activity. The addition of Triton X-100, Tween 20, and Tween 80 showed an activation of Nk up to 141% of its initial activity but SDS strongly inhibited. The enzyme was highly resistant to organic solvents.

**Conclusions:**

Our findings demonstrated that an eight-protease-gene-deficient *Bacillus subtilis* WB800 could overproduce the nattokinase from *B. subtilis* VTCC-DVN-12-01. Due to high resistance to detergents and organic solvents of this nattokinase, it could be potentially applied in organic synthesis and detergent production.

## Background

Nattokinases/Subtilisins (EC 3.4.21.62) belong to the second large family of serine proteases, which share a common property of the catalytic mechanism of the classical Ser*-*His*-*Asp triad situated in a shallow groove on the surface of globular proteins. Nattokinases/Subtilisins are produced in the form of preproenzyme, in which the presequence and prosequence are attached to the N terminus of the mature protein [1]. The presequence functions as a signal peptide for secretion of subtilisin into the external medium. The pro-sequence acts as intramolecular chaperone, guides correct folding of the mature protein and is cleaved by autoproteolysis [2]. The most known nattokinases/subtilisins are secreted by various *Bacillus* species including *B. amyloliquefaciens*, *B. licheniformis*, *B. subtilis*, and *B. amylosacchariticus*.

Due to their widespread distribution, availability and broad substrate specificity, nattokinases/subtilisins gain significant attention and play important role in many biotechnology processes including detergent industry, leather and textile processing, food and feed processing. They can be used as thrombolytic agents directly degrading the fibrin of blood clots, thereby dissolving the thrombi rapidly and completely. Furthermore, these enzymes can catalyze a broad variety of synthetic reactions including synthesis of amino acids and peptide esters [3], and transesterification. Therefore, a number of nattokinases/subtilisins from various *Bacillus* species, especially from *B. subtilis* strains, extensively have been investigated to understand their biochemical and physical properties and to produce at high level for industrial application.

*Bacillus subtilis* serves as an attractive expression host for heterologous protein production because it is nonpathogenic and capable of secreting extracellular proteins directly to the culture medium [4]. For nattokinases/subtilisins, several efforts have been invested to enhance the production of recombinant proteins by elimination of limiting factors, using expression vectors with high structure stability, medium optimization using response surface methodology, and promoter optimization [5]. More efficiently, *B. subtilis* strains were engineered to serve as extracellular-protease-deficient strains for the overproduction of heterologous proteins such as fibrinolytic enzyme/nattokinase/subtilisin in *B. subtilis* WB600 [6-8], xylanase, interleukin 3, staphylokinase in *B. subtilis* WB700 [9-11] and phospholipase C, interleukin 3, xylanase in *B. subtilis* WB800 [10-12].

However, there is no report about high-level expression of nattokinases/subtilisins in *B. subtilis* WB800. For the first time, in this report, we have used *B. subtilis* WB800, which is deficient in eight extracellular proteases [13] to produce the mature nattokinase under the control of *acoA* promoter in large quantities (600 mg/l), which is highest yield of proteins expressed in any extracellular-protease-deficient *B. subtilis* system till date. The recombinant Nk was purified and physicochemical properties were determined.

## Results

### Cloning and sequence analysis of a nattokinase-encoding gene from *B. subtilis*

The gene encoding nattokinase was amplified by PCR with specific primers and sequenced. The 1146-bp insert revealed a complete ORF, predicted to encode a nattokinase (275 aa-mature protein with 27.7 kDa, and a pI of 6.6). Using the SignalP predictions (http://www.cbs.dtu.dk/services/SignalP-2.0/) it revealed that the putative nattokinase had a signal peptide of 30 amino acids [14]. The sequence of the gene nattokinase from *B. subtilis* VTCC-DVN-12-01 showed 99.8-98% identity with corresponding sequences from *B. subtilis* strains (DQ997813, AY940167, K01988, and EF474344), and 81-80% with sequences from *B. amyloliquefaciens* strains (K02496, X00165, and FJ882063). Whereas, the putative amino acid sequence of Nk exhibited 99.7-97.4% identity with the corresponding amino acid sequences from *B. subtilis* strains (ABJ98765, P00783, ACE63521, ADI24411, ABM97611, ACJ06132, and CAE1180) and 87-86% with those from *B. amyloliquefaciens* strains (CAA24990, AAZ66858, and ACS45325). The sequence was deposited in GenBank with an accession number EF061457.

### Expression and purification of nattokinase

**Figure 1:**
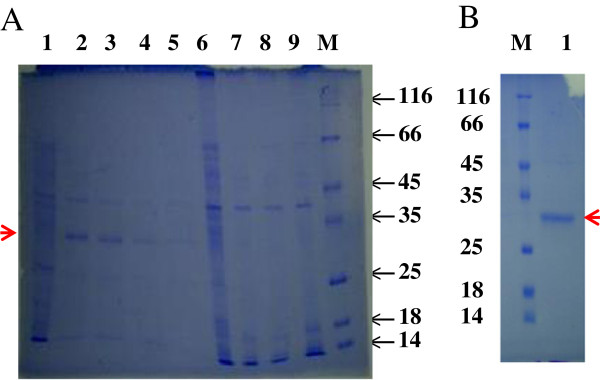
**SDS-PAGE of expressed and purified Nk by ProBond™ Resin. A**. Lane 1–5, culture supernatants; lane 6–9, cell lysates; lane 1,6, WB800/pAC7, lane 2–4, 7–9, WB800/pANk. Lane M, molecular standards indicated in kDa. **B**. Lane 1, purified Nk; Lane M, molecular standards indicated in kDa. The gel was stained by using Coomassie Briliant Blue R250. The band of lane 1 was cut off and used for LC-ESI-MS/MS analysis.

*B. subtilis* WB800/pANk transformants were cultivated in LB medium for the nattokinase production. After acetoin induction for 48 h, the culture supernatants were collected and used for enzyme activity assay. The *B. subtilis* WB800/pANk transformant showing the highest production of nattokinase (100 mg protein per liter, and then 600 mg/l after optimization, data not shown) was used for enzyme production, purification and characterization (Figure 1A). The recombinant nattokinase was purified from the culture supernatant of *B. subtilis* WB800/pANk by affinity chromatography Ni^2+^-ProBond™ resin with a purification factor of 3.25 and a yield of 54.17% (Table 1) and showed a single protein with a molecular mass of approximately 28 kDa on SDS-PAGE (Figure 1), in good agreement with that calculated. In accordance with the purification yield of 54%, the culture supernatant of *B. subtilis* WB800/pANk exhibited the amount of Nk more than one half of total extracellular proteins on SDS-PAGE and most of them was secreted into the culture medium (Figure 1A).

**Table 1:** **Purification steps of the nattokinase from *B. subtilis *WB800/pANk**

**Step**	**Total activity (U)**	**Total protein (mg)**	**Specific activity (U/mg)**	**Purification factor**	**Yield (%)**
Crude nattokinase	24	4.8	5.0	1.0	100
ProBond™ resin	13	0.8	16.25	3.25	54.17

### Identification of recombinant nattokinase

**Figure 2:**
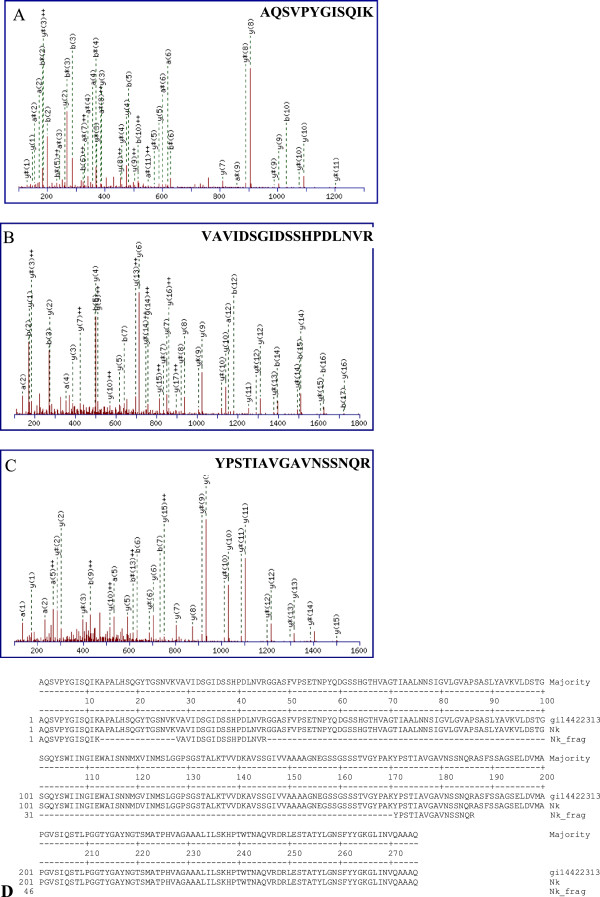
**Monoisotopic mass of three neutral identified peptides. A**. AQSVPYGISQIK position 1–12 (A); **B**. VAVIDSGIDSSHPDLNVR position 28–45; **C**. YPSTIAVGAVNSSNQR position 171–186 found in gi|14422313, nattokinase from *Bacillus subtilis* (GenBank, CAC41625) corresponding to ion scores of 35, 96 and 85 with p < 0.05, respectively. **D**. Alignment of three neutral identified peptides (Nk_Frag) with nattokinase from *B. subtilis* CAC41625 (gi14422313) and putative Nk from *B. subtilis* VTCC-DVN-12-01 (Nk).

The single protein on SDS-PAGE (Figure 1B) was cut out from the gel and used for LC-ESI-MS/MS analysis of mass spectrum database by using Mascot v1.8 program. The total score of nattokinase identification was 566 and matched peptides were 34 fragmentations. Three peptide fragments of the purified enzyme identified by MALDI-TOF mass spectrometry agreed with those of the nattokinase found in GenBank CAC41625 (gi|14422313), nattokinase (*Bacillus subtilis*) AQSVPYGISQIK (position 1–12), VAVIDSGIDSSHPDLNVR (position 28–45), YPSTIAVGAVNSSNQR (position 171–186) (Figure 2A, B, C), corresponding to ion scores of 35, 96, and 85 with p < 0.05, and a monoisotopic mass of 1289.7 Da, 1892.06 Da, and 1662.83 Da, respectively. Whereas the peptide fragments showing ion scores above 42 were identified uniquely or high-similarly with p<0.05. These peptides of the recombinant nattokinase expressed by *B. subtilis* WB800/pANk showed 100% identity with the corresponding fragments of the nattokinase protein from *B. subtilis* (GenBank accession number CAC41625) (Figure 2D).

### Temperature and pH optimum

**Figure 3:**
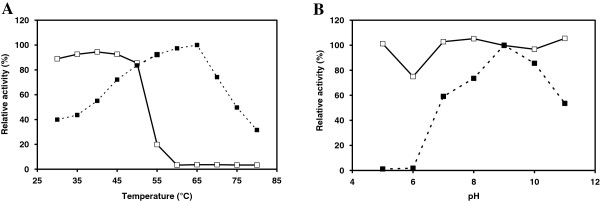
**Effect of temperature and pH. A**. Temperature optimum and stability. **B**. pH optimum and stability. Temperature and pH optimum (■) and stability (□) of Nk from *B. subtilis* VTCC-DVN-12-01. For temperature and pH optimum, the activity of 0.6 μg of Nk was measured with 1 mM Suc-AAPF-*p*NA as a substrate at pH 8 and various temperatures 30-70°C and at 65°C and various pH 5 to 11, respectively. For thermal and pH stability, 0.6 μg of Nk was incubated in 50 mM Na-phosphate buffer at various temperatures 30-70°C for 1 h and in 50 mM acetate (pH 5), phosphate (pH 6–8) and Tris–HCl (pH 9–11) at 30°C for 1 h, respectively. The residual activity was determined at pH 9 and 65°C with 1 mM Suc-AAPF-*p*NA as a substrate.

The optimum temperature of the nattokinase from *B. subtilis* VTCC-DVN-12-01 was 65°C for hydrolysis of Suc-AAPF-*p*NA (Figure 3A). The nattokinase activity increased gradually from 40% (4.5 U/mg) at 30°C to maximum of 100% (11.2 U/mg) at 65°C and then decreased steeply to 74% (8.3 U/mg) at 70°C and 32% (3.5 U/mg) at 80°C. The nattokinase showed maximum activity at pH 9 (12.7 U/mg) (Figure 3B).

### Temperature and pH stability

The nattokinase from *B. subtilis* VTCC-DVN-12-01 was stable up to 50°C and retained more than 85% of its initial activity (Figure 3A) after incubation for 1 h. The enzyme showed a broad pH stability from 5 to 11 and no loss of its initial activity (Figure 3B) except for pH 6 (75% of its initial activity) after incubation for 1 h.

### Effect of EDTA and metal ions

The chelating agent EDTA enveloping metal ions extensively increased the enzyme activity by 18% at the concentration of 1 mM (Table 2) and strongly decreased activity of the enzyme to 21% of its initial activity at the higher concentration of 5–10 mM that means the enzyme might require metal ions for its catalysis. Mg^2+^ activated the nattokinase from *B. subtilis* VTCC-DVN-12-01 with an increase in the activity by up to 62%. Other metal ions Ca^2+^, Cu^2+^, Fe^2+^, Mn^2+^, Ni^2+^, and Zn^2+^ showed a slight effect on the nattokinase activity with an increase by up to 10% and a decrease by up to 27%.

**Table 2:** Effect of metal ions and EDTA on Nk activity

**Additive**	**Remaining activity (%) at concentration (mM) of**		
	**1**	**5**	**10**
Ca^2+^	82 ± 6.71	91 ± 2.94	105 ± 5.46
Cu^2+^	85 ± 1.68	85 ± 3.78	74 ± 2.52
Fe^2+^	101 ± 3.36	103 ± 0.42	104 ± 3.78
Mg^2+^	141 ± 1.68	162 ± 2.10	124 ± 14.69
Mn^2+^	85 ± 1.26	98 ± 2.10	110 ± 5.46
Ni^2+^	91 ± 11.33	105 ± 3.36	105 ± 3.78
Zn^2+^	73 ± 10.91	75 ± 8.39	107 ± 2.52
EDTA	118 ± 6.71	22 ± 0.42	21 ± 0.00

0.6 μg of Nk was incubated in 50 mM Na-phosphate buffer pH 8 at 30°C for 1 h in the presence of 1, 5 or 10 mM of different metal ions. The Nk activity was measured with 1 mM Suc-AAPF-*p*NA as a substrate at pH 9 and 65°C.

### Effect of detergents

The effect of ionic (SDS) and nonionic detergents (Tween 20, Tween 80, and Triton X-100) currently used for denaturing of glycoproteins was tested on the nattokinase activity. The addition of Tween 80, Tween 20, and Triton X-100 at the concentration of 0.2-1% (w/v) showed an activation of the nattokinase from *B. subtilis* VTCC-DVN-12-01 up to 141% of its initial activity (Table 3). However, SDS inhibited the enzyme strongly and decreased the activity up to 11% at the concentration of 0.5-1% (w/v).

**Table 3:** Effect of detergents on Nk activity

**Detergent**	**Remaining activity (%) at concentration (%) of**		
	**0.2**	**0.5**	**1**
Triton X-100	113 ± 0	114 ± 1.2	112 ± 2.7
Tween 20	141 ± 6.2	122 ± 0	118 ± 2.4
Tween 80	138 ± 0.6	139 ± 4.1	138 ± 0.3
SDS	66 ± 3.8	13 ± 1.5	11 ± 0.3

0.6 μg of the Nk was incubated in 50 mM Na-phosphate buffer pH 8 at 30°C for 1 h in the presence of 0.2, 0.5 or 1% (w/v) of various detergents. The Nk activity was measured with 1 mM Suc-AAPF-*p*NA as a substrate at pH 9 and 65°C.

### Effect of organic solvents

Organic solvents are used for solubilizing hydrophobic substrates in enzymatic reactions, thus we have tested effects of various organic solvents. In general, the nattokinase from *B. subtilis* VTCC-DVN-12-01 showed a high resistance to tested organic solvents (Table 4) even the addition of 1-butanol and acetone at the concentration of 10 and 30% (v/v) activated the enzyme to 120-124% of its activity. The addition of 10% (v/v) of ethanol, acetonitrile, and isopropanol increased the enzyme activity by 10-18%.

**Table 4:** Effect of organic solvents on Nk activity

**Solvent**	**Remaining activity (%) at concentration (%) of**	
	**10**	**30**
Methanol	100 ± 10.86	99 ± 3.44
Ethanol	118 ± 1.85	97 ± 2.65
Isopropanol	110 ± 6.36	92 ± 1.32
1-Butanol	120 ± 6.89	124 ± 16.68
Acetonitrile	113 ± 7.15	60 ± 1.59
Acetone	124 ± 4.24	120 ± 5.83

0.6 μg of the Nk was incubated in 50 mM Na-phosphate buffer pH 8 at 30°C for 1 h in the presence of 10 or 30% (v/v) of different organic solvents. The Nk activity was measured with 1 mM Suc-AAPF-*p*NA as a substrate at pH 9 and 65°C.

## Discussion

For the first time, in this report, we have used *B. subtilis* WB800, which is deficient in eight extracellular protease genes (*nprE*, *aprE*, *epr*, *bpr*, *mpr :: ble*, *nprB :: bsr*, *vpr*, *wprA :: hyg*) [13] to produce the mature nattokinase under the control of *acoA* promoter in large quantities (600 mg/l after optimization, data not shown) which is highest yield of proteins expressed in any extracellular-protease-deficient *B. subtilis* system till date (in *B. subtilis* WB800: 1.9 mg/l [15], 5–10 mg/l [16], 100 mg/l [11]; in *B. subtilis* WB700: 260 mg/l [17], 337 mg/l [9]; in *B. subtilis* WB600: 5 mg/l [18], 35.6 mg/l [19]). Most proteins expressed in extracellular-protease-deficient *B. subtilis* system were from different organisms rather than *B. subtilis* except for the nattokinase from *B. natto* expressed in *B. subtilis* WB700 [17]. This answered why the expression level of these proteins were lower than that of the nattokinase from *B. subtilis* VTCC-DVN-12-01. The expression level of the nattokinase from *B. natto* in *B. subtilis* WB700 was 260 mg/l [17], a half of that of Nk from *B. subtilis* VTCC-DVN-12-01 in *B. subtilis* WB800 (600 mg/l). Two reasons might be that firstly, *B. subtilis* WB800 was deficient in one more extracellular protease than *B. subtilis* WB700, and secondly, the production of Nk in *B. subtilis* WB800 was optimized (data not shown).

The optimal pH and temperature values for the nattokinase from *B. subtilis* VTCC-DVN-12-01 were observed at pH 9 and 65°C, respectively, somehow higher than those for the nattokinases from *B. subtilis* natto B-12 [20], *B. subtilis* TKU007 [21], and *B. subtilis*[22] (pH 8–9 and 37-40°C), for the subtilisins from *B. amyloliquefaciens* DC-4 [7] and *B. stearothermophilus*[23] (pH 9 and 48-60°C), and for the proteases from *B. subtilis*[24] and *B. licheniformis*[25] (pH 6.5-9, 40-47°C).

The thermal (up to 50°C) and pH stability (at pH 5 to 11) for Nk from *B. subtilis* VTCC-DVN-12-01 were in agreement with those from other reports. The nattokinase from *B. subtilis* natto B-12 showed high thermostability at temperatures from 30 to 50°C and alkaline stability within the range of pH 6–9 [20]. The nattokinase BSN1 from *B. subtilis* TKU007 showed pH stability at pH 4–11 and thermal stability less than 50°C [21]. The subtilisin J from *B. stearothermophilus* retained up to 80% of its activity at pH 6 to 11 after 24 h incubation at 4°C and 1 h incubation at 40°C in the presence of 2 mM CaCl_2_[23]. The enzyme retained about 50% of its activity even after treatment at 60°C for 30 min in the presence of 2 mM CaCl_2_.

Nk from *B. subtilis* VTCC-DVN-12-01 was activated by the addition of Mg^2+^ as the alkaline protease from *B. magaterium*[26] and *B. subtilis* MTTC N0-10110 [27]. The nattokinase from *B. subtilis* natto B-12 was activated by Zn^2+^ and obviously inhibited by Fe^3+^ and Al^3+^[20]. The alkaline protease from *B. magaterium* was activated by Mn^2+^, Ca^2+^, and Mg^2+^ and inhibited by EDTA at 1 mM [26]. The activity of the alkaline protease produced by *B. subtilis* MTTC N0-10110 also showed an activation by Mg^2+^ but a slight inhibition by Zn^2+^ at 5 mM [27].

The effect of detergents on Nk from *B. subtilis* VTCC-DVN-12-01 was in agreement with current observations that the addition of Tween 20 and Tween 80 at 1% to the alkaline protease from *Bacillus* SB5 increased the activity by 22 and 36%, respectively, after incubation for 0.5 h and by 44 and 68% after incubation for 1 h at 40°C, whereas SDS decreased the activity by 12 and 40% after incubation for 0.5 h and 1 h, respectively [28].

Subtilisins/Nattokinases have been used to catalyze the peptide synthesis and transesterification in organic solvents [29-31]. Thus many studies were reported on the improvement in catalytic activity using organic solvents to expand their use in organic synthetic applications of the wild-type and mutant subtilisins. Chen and Arnold (1991) used random mutagenesis to enhance activity of subtilisin E from *B. subtilis* in polar organic media: the triple mutant D60N+Q103R+N218S was 38 times more active than the wild-type subtilisin E in 85% DMF [32]. Takagi *et al*. (2000) engineered the subtilisin E from *B. subtilis* 168 for enhanced stability and activity in polar organic solvents. The Cys170/Cys195 mutant subtilisin E was found to be more stable in polar organic solvents, such as dimethylformamide and ethanol, than the wild-type enzyme, even under reducing conditions. The amino-terminal engineered disulfide bond (Gly61Cys/Ser98Cys) and the mutation Ile31Leu were introduced to enhance the stability and catalytic activity. A prominent 3-4-fold increase in the catalytic efficiency occurred in the quintet mutant enzyme over the range of dimethylformamide concentration (up to 40%) [33]. Dimethyl sulfoxide, acetone, and branched alcohols were found to be the most benign solvents for wild-type and mutant subtilisin-(Carlsberg and BPN’)-catalyzed hydrolyses [34], whereas dioxane, tetrahydrofuran, and *N*-methyl-2-pyrrolidinone seriously reduced catalytic activities, even at low concentrations. Our wild-type nattokinase from *B. subtilis* VTCC-DVN-12-01 possessing a property of high resistance to organic solvents including acetone and alcohols, has a potential use in organic synthetic applications without any modification of the enzyme.

The coding sequence of Nk from *B. subtilis* VTCC-DVN-12-01 exhibited similarities of 99.6%, 85.8% and 70.1% with the corresponding amino acid sequences of subtilisin E from *B. subtilis* 168, Carlsberg from *B. licheniformis* and subtilisin BPN’ from *B. amyloliquefaciens*. However, the mentioned-above amino acids (60, 103, 218 in subtilisin E [32], 31, 61, 98, 170, 195 in subtilisin E from *B. subtilis* 168 [33] and 166, 222 in subtilisin Carlsberg and BPN [34]), whose mutations increased stability, were maintained in Nk. It demonstrated that the property of high resistance to organic solvents of Nk from *B. subtilis* VTCC-DVN-12-01 was not due to those amino acids, whose mutations increased stability, but might be due to other amino acids, different from the wild-type subtilisin E, Carlsberg and BPN. On the other hand, in accordance with our results, Bonneau *et al.* (1993) also found that acetone, and branched alcohols were the most benign solvents for wild-type and mutant subtilisin-catalyzed hydrolyses.

## Conclusions

A nattokinase from *Bacillus subtilis* VTCC-DVN-12-01 was overproduced by using an eight-protease-gene-deficient *Bacillus subtilis* WB800. The enzyme showed high resistance to detergents and organic solvents. The biochemical properties of this nattokinase make it possible to be used in organic synthesis and detergent production.

## Materials and methods

### Chemicals and reagents

*N*-succinyl-Ala-Ala-Pro-Phe-*p*-nitroanilide (Suc-AAPF-*p*NA) was purchased from Sigma-Aldrich Co. (St. Louis, USA). Triton X-100 was from Fluka™, a Sigma-Aldrich Co. (St. Louis, USA). Tween 20, Tween 80, peptone, yeast extract, and casamino acid were provided by Bio Basic Inc. (Ontario, Canada). Restriction enzymes, T4 ligase, *Taq* and *Pfu* polymerase were supplied by Fermentas (Thermo Fisher Scientific Inc., Waltham, USA). DNA Gel-Extraction Kit was from Promega (San Luis Obispo, CA, USA). ProBond™ Resin was from Invitrogen Corp. (Carlsbad, USA). Primers were provided by Bioneer (Daejeon, Korea). All other reagents were of analytical grade unless otherwise stated.

### Bacterial strains and expression plasmids

**Figure 4:**
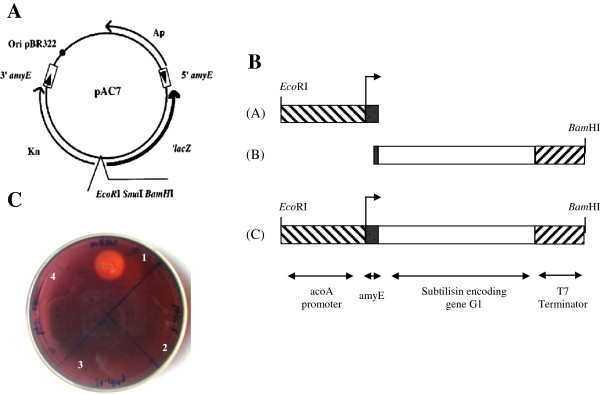
**Construction of expression plasmid. A**. Vetor pAC7 map. **B**. Construction of expression cassette *acoA-nk-T7*. Fragment A: *acoA* promoter plus amyE, fragment B, subtilisin encoding gene plus T7 terminator; fragment C, *acoA* promoter + amyE + *nk* + T7 terminator. **C**. Starch hydrolysis of *B. subtilis* WB800 (1), the transformants *B. subtilis* WB800/pANk (2-3) and WB800/pAC7 (4).

The bacterial strain *Bacillus subtilis* VTCC-DVN-12-01 (deposited at Vietnam Type Culture Collection, Institute of Microbiology and Biotechnology, Vietnam National University in Hanoi) was used as the source of the nattokinase (*nk*) gene. *Escherichia coli* DH5α (F–, ø80d*lacZ*ΔM15, Δ(*lacZYA-argF*)U169, *deo*R, *recA*1, *endA*1, *hsd*R17(rk^–^, mk^+^), *phoA*, *supE*44, λ^–^, *thi*-1, *gyrA*96, *relA*1), pTZ57R/T and pJET1.2/blunt vector (Fermentas, Thermo Fisher Scientific Inc., Waltham, USA), pET22b+ (Novagen, Merck KGaA, Darmstadt, Germany) and pMSE3 (Institute of Biochemistry, Greifswald University) were used for DNA manipulations and amplification. *Bacillus subtilis* strain WB800, which is deficient in eight extracellular proteases (*nprE*, *aprE*, *epr*, *bpr*, *mpr :: ble*, *nprB :: bsr*, *vpr*, *wprA :: hyg*), was used as a host for expression of *nk* gene (Institute of Biochemistry, Greifswald University). pAC7 plasmid (Figure 4A) with 10.6 kb in size contains the ampicillin (Ap^r^) and kanamycin (Kn^r^) resistance gene for *E. coli* and *Bacillus*, respectively, promoterless *lacZ* and *5′amyE-3′amyE*. The multiple cloning site containing *Eco*RI, *Sma*I, and *Bam*HI was placed between the kanamycin resistance gene and *lacZ*. In pAC7 plasmid transformation, the *Kn*-*lacZ* region was integrated into the chromosomal locus *amyE* in *B. subtilis* and thus led to an α-amylase deficient phenotype [35]. Luria-Bertani medium (LB) containing 1% (w/v) bacto tryptone, 0.5% (w/v) yeast extract, 1% (w/v) NaCl, at a pH of 7–7.5 was used for the cultivation of *E. coli* and *B. subtilis*. LB agar contained additionally 2% (w/v) agar and 100 μg ampicillin/ml or 25 µg kanamycin/ml.

### DNA manipulations

Genomic and plasmid DNA isolation was carried out by the method as previously described [36]. DNA fragments and PCR products were excised from a 0.8% agarose gel and purified by a gel extraction kit (Qiagen, Venlo, The Netherlands) according to manufacturer’s instruction. DNA sequencing was performed on ABI PRISM 3100 Avant Genetic Analyzer (Applied Biosystems Inc., Foster City, USA). *E. coli* DH5α cells were transformed using heat shock method as previously described [36].

### DNA amplification and plasmid construction

Based on the nucleotide sequence of the gene encoding subtilisin from *B. subtilis* 168, two oligonucleotides NkF and NkR (Table 5) were designed as primers to amplify the putative gene *nk* from *B. subtilis* VTCC-DVN-12-01 with the introduction of *Eco*RI and *Xho*I restriction site at 5’ of the forward and reverse primer, respectively. The PCR mixture contained 2.5 μl 10× PCR buffer, 2 μl of 2.5 mM dNTP, 2 μl of 25 mM MgCl_2_, 1 μl genomic DNA (50–100 ng), 0.2 μl 5 unit *Taq* polymerase and 1 μl each primer (10 pmol), supplemented with 15.3 μl distillated water to a final volume of 25 μl. The thermocycler conditions were performed as follows: 94°C/4′; 35 cycles of 94°C/45″, 54°C/1′, 72°C/1′; 72°C/10′. The PCR products amplified from the genomic DNA with both primers NkF and NkR were inserted into the cloning vector pTZ57R/T, resulting in pTNk and sequenced. It was followed by ligation of the *Eco*RI-*Xho*I digested pTNk products with pET22b+ linearized by the same enzymes, resulting in pENk under the control of the T7-promoter and T7-terminator.

**Table 5:** **Primers used for construction of expression cassette *acoA-nk-T7***

**Primer**	**Sequence 5′→3′**	**PCR product**
NkF	GC *GAATTC* GC GTGAGAAGCAAAAAATTG	*nk*
NkR	GC *CTCGAG* TTGTGCAGCTGCTTGTAC	
*acoA*F	GC *GAATTC* TCAGTCAAACGATGCAG	Fragment A
*acoA*R	AGC GCT CGC AGC CGC CGG TCC TGC	
*acoA*-NkF	GAC CGG CGG CTG CGA GTG CTg tga gaa gca aaa aat tgt g	Fragment B
T7R	GC *GGATCC* CAA AAA ACC CCT CAA GA	

The expression plasmid pANk was constructed from pAC7 (Figure 4A) and the expression cassette consisted of *acoA* promoter - nattokinase gene from *B. subtilis* VTCC-DVN-12-01 - T7 terminater (*acoA*-*nk*-T7) (fragment C, Figure 4B), which was achieved by a fusion PCR from two DNA fragments A and B (Figure 4B). The DNA fragment A containing *acoA* promoter from the *acoABCL* operon plus *amyE* α-amylase signal sequence from *B. subtilis*[37] (Figure 4B) was amplified from the pMSE3 vector as template with two primers *acoA*F and *acoA*R (Table 5) with the introduction of the underlined *Eco*RI restriction site at 5′ of the forward primer. The PCR mixture contained 2 μl of 10× PCR buffer, 1.5 μl of 25 mM MgSO_4_, 2 μl of 2.5 mM dNTP, 0.5 μl of 2.5 U/μl *Pfu* polymerase, 1 μl of each primer (10 pmol), 1 μl of pMSE3 (10 ng), and 11 μl H_2_O. The thermocycler conditions were performed as follow: 95°C/4′; 35 cycles of 95°C/45″, 53°C/45″, 72°C/45″; and 72°C/10′.

The DNA fragment B containing the nattokinase encoding gene *nk* and T7 terminator (Figure 4B) was amplified from pENk plasmid as template with two primers *acoA*-NkF and T7R (Table 5), which contained a complementary fragment with *amyE* α-amylase signal sequence and a *Bam*HI site at 5′ of the forward and reverse primer, respectively (Figure 4B). The PCR mixture contained 2 μl of 10× PCR buffer, 1.5 μl of 25 mM MgSO_4_, 2 μl of 2.5 mM dNTP, 0.5 μl of *Pfu* polymerase (2.5 U/μl), 1 μl of each primer (10 pmol), 1 μl of pENk (10 ng), 11 μl H_2_O. The thermocycler conditions were performed as above mentioned.

The two PCR products were fused in a fusion PCR mixture contained 2 μl of 10× PCR buffer, 2 μl of 25 mM MgSO_4_, 2 μl of 2.5 mM dNTP, 0.5 μl of 2.5 U/μl *Pfu* polymerase, 2 μl of DNA fragment A (100 ng), 2 μl of DNA fragment B (100 ng), and 9.5 μl H_2_O. The thermocycler conditions were performed as follow: 95°C/4′; 15 cycles of 95°C/45″, 56°C/1′30″, 72°C/1′45″; 72°C/10′. Subsequently, the fusion PCR products were amplified with primers *aco*AF and T7R in a PCR mixture containing 2 μl of 10× PCR buffer, 1.5 μl of 25 mM MgSO_4_, 2 μl of 2.5 mM dNTP, 0.5 μl of 2.5 U/μl *Pfu* polymerase, 1 μl of each primer (10 pmol), 2 μl of the fusion PCR product, 10 μl H_2_O and the thermocycler conditions: 95°C/4′; 35 cycles of 95°C/45″, 53°C/45″, 72°C/1′45″; 72°C/10′.

The fusion PCR products were inserted into a pJET1.2/blunt vector, resulting in pJ*acoA-nk*-T7. It was followed by ligation of the *Eco*RI-*Bam*HI digested *acoA-nk*-T7 products with pAC7 linearized by the same enzymes, resulting in the recombinant plasmid pANk under the control of the *acoA*-promoter induced by acetoin and possessing the ampicillin marker. *B. subtilis* WB800 cells were transformed according to the method as previously described [38] with the control plasmid pAC7 and expression plasmid pANk, resulting in expression strains WB800/pAC7 and WB800/pANk, respectively, where *Kn*-*lacZ* regions integrated into the genomic locus *amyE*. These two strains were kanamycin resistant and α-amylase-deficient and did not show α-amylase activity on LB-agar plate containing 0.5% (w/v) starch (Figure 4C; 1, WB800; 2-3, WB800/pANk, 4, WB800/pAC7). The integration of the fusion construct was also confirmed by PCR using the genomic DNA from the strain WB800/pANk with the primers *acoA*F and T7R.

### Gene expression

For expression of Nk in *B. subtilis* WB800/pANk, 2.5 ml of an overnight culture were inoculated into 250 ml LB medium in a 1-liter Erlenmeyer flask and grown at 37°C with agitation at 200 rpm. The culture was cultivated until an optical density (OD) at 600 nm reached 1.5 (approximately at the end of the exponential growth) and then induced by the addition of acetoin to a final concentration of 0.5% (w/v). After the acetoin induction for 48 h, the culture supernatant containing the extracellular recombinant nattokinase was harvested.

### Purification of nattokinase

The fusion form Nk carrying a C-terminal 6xHis tag was expressed in *B. subtilis* WB800 and purified using affinity chromatography with Ni^2+^-ProBond™ resin (Invitrogen Corp., Carlsbad, USA) under native conditions. A volume of 8 ml culture supernatant in LB medium was harvested by centrifugation at 8000 rpm and 4°C for 5 min, and was applied to a column containing 2 ml resin which was equilibrated with native binding buffer and incubated for 60 min at room temperature with gently inverting several times. The resin in column was settled by gravity and the supernatant was carefully aspirated to remove all non-6xhis-tagged proteins and washed three times with 8 ml native wash buffer. The bound protein was eluted with 8 ml of native elution buffer and 1 ml per fraction was collected.

### MALDI-TOF mass spectrometry

The nattokinase was identified by MALDI-TOF mass spectrometry as previously described [39]. The predicted protein band on SDS-PAGE was cut out and the target protein was digested by trypsin into small peptide fragments. The mixture of peptides was analyzed on nano-LC liquid chromatography and ionized by the ESI (electrospray ionization). The mass spectra were obtained on the QSTAR® XL mass spectrometer (Applied Biosystems, MDS SCIEX, Canada) with a nano-ESI ion source. Protein fragments were identified by the Mascot v1.8 Search Software from the database (NCBInr, SwissProt). Peptide fragments showing ion scores above 42 were identified uniquely or high-similarly with p<0.05.

### Nattokinase activity assay

The reaction mixture (200 μl) contained 50 mM Tris–HCl (pH 8.0), 5 mM CaCl_2_ and 1 mM Suc-AAPF-*p*NA as a substrate. After adding 3 μl of the enzyme (0.6 μg) and incubating the mixture at 60°C for 5 min, the enzyme reaction was stopped by the addition of 50 μl of 30% (v/v) acetic acid. The amount of *p*-nitroanilide released from the substrate was determined from the absorption at 410 nm with the molar absorption coefficient value of 8900 M^-1^ cm^-1^[40] on an automatic UV spectrophotometer UV-2500 (LaboMed Inc., Culver City, CA, USA). One unit of enzymatic activity was defined as the amount of the enzyme that released 1 μmol of *p*-nitroanilide per min under experimental conditions. The specific activity was defined as the enzymatic activity per milligram of protein.

### Electrophoresis analysis and protein concentration

The homogeneity and molecular mass of the nattokinase was determined by 12.5% SDS polyacrylamide gel electrophoresis [41] with Biometra equipment (Göttingen, Germany). Proteins were visualized by staining with 0.1% (w/v) Coomassie Brilliant Blue R-250. Protein concentrations were estimated by the method of Bradford with the bovine serum albumin as standard [42].

### Temperature and pH optimum

The temperature and pH optimum of the nattokinase were determined by measuring the activity, as described above, using 50 mM Tri-HCl buffer pH 8 at the temperature range of 30 to 80°C, and using 50 mM acetate buffer (pH 4–5), 50 mM phosphate buffer (pH 6–8), and 50 mM Tris–HCl buffer (pH 9–11) at 65°C, respectively.

### Temperature and pH stability

For the determination of temperature and pH stability, the purified enzyme, 0.6 μg for each reaction, was preincubated at the temperature range of 30 to 80°C and pH 7 for 1 h, and under various pH conditions (with 50 mM acetate at pH 4–5, 50 mM phosphate at pH 6–8, and 50 mM Tris buffer at pH 9–11) at 30°C for 1 h, respectively. The residual activity was determined at pH 9 and 65°C.

### Effect of metal ions, detergents and organic solvents

The effect of metal ions, EDTA, detergents, and organic solvents on the nattokinase activity was determined by preincubating the enzyme, 0.6 μg for each reaction, in 50 mM sodium phosphate buffer pH 7 in the presence of 1, 5, and 10 mM of metal ions (Ca^2+^, Cu^2+^, Fe^2+^, Mg^2+^, Mn^2+^, Ni^2+^, Zn^2+^) or EDTA, or in the presence of 0.2, 0.5, 1% (w/v) of detergents, or in the presence of 10 and 30% (v/v) of organic solvents at 30°C for 1 h. The residual activity was determined at pH 9 and 65°C.

All measurements were carried out in triplicate with the resulting values being the mean of the cumulative data obtained.

### DNA and amino acid sequence alignments

Sequence alignments were constructed and analyzed using the program MegAlign DNAStar. The signal peptide was predicted using SignalP 4.0 Server (http://www.cbs.dtu.dk/services/SignalP/) [14].

## Abbreviations

Gene nk: Gene encoding nattokinase from *Bacillus subtilis* strain VTCC-DVN-12-01; Protein/Enzyme Nk: Nattokinase from *Bacillus subtilis* strain VTCC-DVN-12-01.

## Competing interests

The authors declare that they have no competing interests.

## Authors’ contributions

TDQ designed the experimental setup, initiated the project, assisted with data analysis and manuscript preparation, read and approved the final manuscript. TTN prepared manuscript and together with HTL performed experiments of plasmid construction, expression, purification and characterization of the nattokinase. All authors read and approved the final manuscript.
